# Fast Track Open Partial Nephrectomy: Reduced Postoperative Length of Stay with a Goal-Directed Pathway Does Not Compromise Outcome

**DOI:** 10.1155/2008/507543

**Published:** 2008-09-04

**Authors:** Bilal Chughtai, Christa Abraham, Daniel Finn, Stuart Rosenberg, Bharat Yarlagadda, Michael Perrotti

**Affiliations:** Urology Service, Saint Peter's Cancer Care Center, and Department of Surgery, Albany Medical College, Albany, NY 12208, USA

## Abstract

*Introduction*. The aim of this study is to examine the feasibility of reducing postoperative hospital stay following open partial nephrectomy through the implementation of a goal directed clinical management pathway. *Materials and Methods*. A fast track clinical pathway for open partial nephrectomy was introduced in July 2006 at our institution. The pathway has daily goals and targets discharge for all patients on the 3rd postoperative day (POD). Defined goals are (1) ambulation and liquid diet on the evening of the operative day; (2) out of bed (OOB) at least 4 times on POD 1; (3) removal of Foley catheter on the morning of POD 2; (4) removal of Jackson Pratt drain on the afternoon of POD 2; (4) discharge to home on POD 3. Patients and family are instructed in the fast track protocol preoperatively. Demographic data, tumor size, length of stay, and complications were captured in a prospective database, and compared to a control group managed consecutively immediately preceding the institution of the fast track clinical pathway. *Results*. Data on 33 consecutive patients managed on the fast track clinical pathway was compared to that of 25 control patients. Twenty two (61%) out of 36 fast track patients and 4 (16%) out of 25 control patients achieved discharge on POD 3. Overall, fast track patients had a shorter hospital stay than controls (median, 3 versus 4 days; *P* = .012). Age (median, 55 versus 57 years), tumor size (median, 2.5 versus 2.5 cm), readmission within 30 days (5.5% versus 5.1%), and complications (10.2% versus 13.8%) were similar in the fast track patients and control, respectively. *Conclusions*. In the present series, a fast track clinical pathway after open partial nephrectomy reduced the postoperative length of hospital stay and did not appear to increase the postoperative complication rate.

## 1. INTRODUCTION

Radical nephrectomy has considered the optimal surgical approach to the management of renal cancer [1]. Additional options include observation, cryosurgery, and radiofrequency ablation [2, 3]. Management of the small renal mass requires multiple perspective decision making on the part of the physician [4]. Recently, partial nephrectomy has been shown to have efficacy in the management of select patients with small renal masses (4 cm or less) [5, 6]. Several investigators have reported an expanded use of this approach with success in patients with larger renal neoplasms (7 cm or less). Hospital stay after partial nephrectomy is usually 5–8 days [7]. Factors limiting early discharge are usually pain, stress-induced major organ dysfunction (i.e., ileus, atelectasis), tradition, fatigue, pain, nausea, and morbidity [8, 9]. In other abdominal procedures, including colon resection, lung transplant, and laparoscopic nephrectomy, the introduction of a program comprised of optimized pain relief using nonsteroidal anti-inflammatories for analgesia enforced oral nutrition and mobilization, and revision of traditional care principles has reduced hospital stay from 5–8 days to 220 133 days in some studies [10, 11]. The concept of fast-track (FT) surgery has recently attracted more interest, but has not yet been applied in patients undergoing partial nephrectomy. The purpose of this study was to investigate the postoperative course before and after the introduction of a fast track program in patients undergoing open partial nephrectomies. We sought to determine whether the use of this fast track program might decrease length of hospital stay without sacrificing outcomes.

## 2. MATERIALS AND METHODS

A fast track clinical pathway for open partial nephrectomy was introduced at our institution in July 2006. The pathway has an established management protocol (Table 1). All patients undergoing open partial nephrectomy from July 2006 thus far at our institution were managed by the fast track protocol, and comprise the study cohort. Patients undergoing laparoscopic partial nephrectomy and robotic partial nephrectomy were not included. In the event that the decision was made intraoperatively to perform complete nephrectomy, patients were included on an intention-to-treat basis. Demographic data, tumor size, blood loss, transfusion, final pathology, margin status, length of hospital stay, and complications were captured in a prospective database, and compared to a control group managed consecutively immediately preceding the institution of the fast track clinical pathway.** All operations were performed for a renal tumor less than 7 cm in greatest diameter by the same surgical team.

### 2.1. Preoperative preparation

Patients were admitted to the hospital on the day of surgery and performed all preparation on an outpatient basis. All patients were counseled preoperatively regarding the target goals outlined in Table 1. Aspirin and nonsteroidal anti-inflammatory drugs (NSAIDs) were stopped 10–14 days prior to surgery. Coumadin was stopped 5 days preoperatively and stat protime/partial thromboplastin time obtained on the morning of surgery to confirm acceptable value. All patients received 3 bisacodyl tablets and 1 bottle of magnesium citrate at noon on the day prior to surgery. All patients received erythromycin and neomycin antibiotic per oral (PO), 500 mg (milligram) tab, at 3 pm, 6 pm, and 9 pm on the day prior to surgery. All patients were instructed to eat lightly and orally hydrate on the day prior to surgery.

### 2.2. Intraoperative

This report is restricted to patients undergoing open partial nephrectomy on an intention to treat basis. General anesthesia, oral gastric tube (removed at case conclusion), and Foley catheter were used in all cases. Compression pneumatic stockings were placed as soon as the patient moved from the stretcher onto the operating table. The retroperitoneal flank approach was used. All patients were positioned with the bean bag, with legs straight, hyper-extended with the kidney rest raised maximally. Before draping, the surgeon marked the posterior axillary line (PAL), the anterior axillary line (AAL), the lateral border of the rectus muscle, and the course of the 10th, 11th, and 12th ribs. Incision was made from the PAL to the AAL in the course of the 11th rib using the cautery on pure cut (setting of 30). The distal tip of the 11th rib was removed in the standard manner.** An extra-pleural/extra-peritoneal approach to the kidney was used. The kidney was explored, and vessel loop passed to tag the ureter, renal artery, and renal vein. A double loop was passed around the vein for subsequent occlusion. Patients received mannitol 12.5 gm (gram) IV (intravenous) bolus prior to manipulation of the renal vessels followed by 5 gm/hour continuous infusion for the remainder of the operation. Renal artery was clamped in all cases and the kidney cooled. The renal vein was selectively occluded as required to provide a bloodless operative field. The collecting system was closed with 3–0 monocryl on SH needle in all cases. Renal arteries and venules were oversewn in figure of eight fashions with 3–0 monocryl on SH needle. Prior to the removal of the renal artery clamp, the kidney was reconstructed essentially obliterating the resection defect utilizing 0-Chromic suture on CT needle in horizontal mattress fashion. This resulted in a reniform shape approximation in nearly all cases. The rib bed and skin were infiltrated with 0.25% marcaine (30 ml (milliliters)). The skin wound was closed in a subcuticular (3–0 monocryl). A *#*7 Jackson-Pratt drain was placed in all cases. In no case was a ureteral stent placed.

### 2.3. Postoperative

On POD 0 (postoperative day), on the evening of surgery, patients received celecoxib 200 mg per oral in the postanesthesia care unit (PACU) with sip when awake, and daily thereafter (Table 1). Morphine sulfate was administered IV at 2 hour intervals as needed. Metoclopramide was administered IV 10 mg every 6 hours. Famotidine was administered 20 mg IV every 12 hours. On the day of surgery, patients ambulated and were encouraged to take liquids by mouth.** On POD 1, diet was advanced to tray of clears, and oral pain medication administered (hydrocodone/acetominophen 5–10 mg/500 mg every 4 hours as needed). On day 2, the Foley catheter was removed at 7 am and regular diet initiated.** Patients received milk of magnesia 30 cc PO at 8 am and a repeat dose in 4 hours.** Jackson-Pratt drain fluid was sent to the laboratory for creatinine measurement and if equal to serum creatinine level, the Jackson-Pratt drain was removed. The patient was assessed and discharged to home on POD 3 if appropriate.

## 3. RESULTS

A total of 33 patients were managed by fast track and compared to 25 control patients (Table 2). The estimated blood loss, transfusion rate, tumor size, pathology, and complication rate were similar between groups. There was, however, a significant difference in the length of hospital stay observed between groups. Of 25 control patients, 4 (16%) achieved discharge to home in <3 days compared to 22 (67%) of the 33 patients managed in the fast track program.** Overall, fast track patients had a shorter hospital stay compared to controls (median, 3 days versus 4 days; *P* = .012). Of the 11 patients in the fast track cohort who were discharged after the third postoperative day, this was due to poor ambulation/inadequate pain control (*n* = 5), abdominal bloating (*n* = 3), multiple co-morbidities (*n* = 2), and respiratory distress postoperatively requiring ICU care (*n* = 1).

Complications are worth noting to determine whether the fast track approach was harmful in any way to the study cohort. In the control cohort, there was 1 patient with respiratory distress requiring ICU admission, 3 patients received blood transfusion, there were 2 conversions to complete nephrectomy, and 1 positive surgical margin. In the fast track cohort there was 1 patient with respiratory distress requiring ICU admission, 2 patients required blood transfusion, there was one conversion to total nephrectomy, 1 postoperative bleed (gross hematuria) requiring selective arterial embolization, and 1 urine leak requiring percutaneous drainage and ureteral stent placement.

The percentage of patients with malignancy increased in the fast track cohort compared to control (85% versus 76%). This may represent improved preoperative assessment.

## 4. DISCUSSION

Since 1950 in the United States, there has been a 126% increase in the incidence of renal cancer [7, 8]. Although there has been an increase in all stages of renal cancer including advanced cases (i.e., regional extension, distant metastases), there has been the greatest increase in those discovered incidentally [8, 12]. In the early 1970s, approximately 10% of tumors were detected incidentally compared with 61% in 1988 [8].

Previous studies of other types of major surgery have shown that a combined effort comprising intensive preoperative information, effective postoperative pain relief and enforced mobilization, and early enteral nutrition can accelerate postoperative recovery and decrease hospital stay [11, 13].

Investigators have recently illustrated that in elderly high-risk patients undergoing colonic resection, mean hospital stay could be reduced to 2-3 days [11]. In another group of high risk patients undergoing open aortic surgery, mean hospital length of stay was reduced from a mean of 9 days to 5 days [14–16]. In a study by Harinath et al., a decrease in length of stay was observed from 5 to 4 days for ileal pouch-anal anastomosis [11]. The concept of fast track has also been applied to infants and children [17]. In the urologic literature, after radical prostatectomy, median hospital stay in 252 consecutive patients was reduced to 1 day [13]. Specifically with kidney surgery, postoperative hospital stay after open nephrectomy was reduced to 4 days [18]. With laparoscopy, hospital stay has been reduced to 2 days with an FT rehabilitation program [19].

In this study, with the introduction of a fast-track program, open partial nephrectomy hospital stay was decreased to 3 days, compared to 4 days before implementation of the program. Sixty six per cent of patients achieved a target discharge on day 3 or less.** Notably, both groups did have similar characteristics as demonstrated in Table 2. The estimated blood loss, transfusion rate, tumor size, pathology, and complication rate were similar between groups. We suspect that based upon our data the main contributing factors responsible for the decrease in hospital stay was a clear protocol of expectations at each stage of the recovery period. This was accomplished in the present series without an apparent increase in complication rate. Most notably, fast track did not lead to an increase in readmissions.

It is impossible to discern exactly which components of our protocol are “more essential” than others, and this would require selective application in future investigations. In addition, we have no proof from the present investigation that the fast track protocol is advantageous to the patient. The purpose of the present study was to assess the feasibility of such an approach and we conclude that such an approach is feasible. Ultimately, the patients in this study decided their discharge date. Most patients were eager to receive discharge to home as soon as they are medically safe. One patient in the fast track protocol was discharged to home at her request on POD 2. She expressed regret at doing so at her first postoperative visit.

Our readmissions to the hospital were as follows. One (3.0%) patient had returned to hospital for postoperative hemorrhage resulting in gross hematuria. This patient was managed successfully with embolization. One (3.0%) patient had returned to hospital forurinoma. This was managed successfully with indwelling ureteral stent for 6 weeks and transient percutaneous drainage of urinoma. Both patients had complex resections, and it is unlikely that fast track management resulted in return to hospital.

## 5. CONCLUSIONS

In the present investigation, a fast track clinical pathway after open partial nephrectomy reduced the postoperative length of hospital stay and did not appear to increase the postoperative complication rate.

## Figures and Tables

**Table 1:** Fast track pathway.

Day	Goal
Preoperative	Patient and family counseling
	Medication review
	Preparation instructions

Postoperative day 0 (evening of surgery)	OOB at least once
	Clear liquids

Postoperative day 1	OOB four times
	Liquid diet
	Oral pain medications

Postoperative day 2	OOB ad lib
	Regular diet
	Remove Foley catheter at 7 am
	Flank drain fluid for Cr at 2 pm
	Remove drain if fluid Cr = serum Cr

Postoperative day 3	Discharge to home

**Table 2:** Outcomes of fast track open partial nephrectomy.

	Conservative group	Fast track group
(N)	25	33
Discharge in <3 days	4	22
Age range	32–74	39–73
Male/female	18/8	22/11

Length of stay		

Range	3–10 days	2–6 days
Median	4 days	3 days
Average	4.4 days	3.3 days

Estimated blood loss		

Range	50–500 cc	50–600 cc
Median	200 cc	200 cc
Average	228 cc	263 cc

Transfusions	3	2

Complications	4	4

Respiratory distress	1	1
Conversion to nephrectomy	2	1
Post operative bleed	0	1
Urine leak	0	1

Tumor size		

Range	1.1–6.8 cm	1.2–6.2 cm
Median	2.5 cm	2.5 cm
Average	2.8 cm	2.9 cm

Pathology		

Clear Cell RCC	17 (68%)	25 (76%)
Papillary RCC	2 (8%)	3 (9%)
Chromophobe RCC	—	3 (9%)
Oncocytoma	3 (12%)	—
AML	1 (4%)	2 (6%)
Other	2 (8%)	—
